# Does Distance Matter? Metabolic and Muscular Challenges of a Non-Stop Ultramarathon with Sub-Analysis Depending on Running Distance

**DOI:** 10.3390/nu17233801

**Published:** 2025-12-04

**Authors:** Lucas John, Moritz Munk, Roman Bizjak, Sebastian V. W. Schulz, Jens Witzel, Harald Engler, Christoph Siebers, Michael Siebers, Johannes Kirsten, Marijke Grau, Daniel Alexander Bizjak

**Affiliations:** 1 Department of Internal Medicine, Division of Sports and Rehabilitation Medicine, University Hospital Ulm, 89075 Ulm, Germany; moritz.munk@uni-ulm.de (M.M.);; 2 Department of Central Informatics, Division of Applications and Databases, University of Zurich, CH-8006 Zurich, Switzerland; 3 Institute of Medical Psychology and Behavioral Immunobiology, Center for Translational Neuro- and Behavioral Sciences, University Hospital Essen, University of Duisburg-Essen, 45147 Essen, Germany; 4 Institute of Forensic Psychiatry and Sex Research, Center for Translational Neuro- and Behavioral Sciences, University of Duisburg-Essen, 45147 Essen, Germany; 5 Institute for Cardiovascular Research and Sports Medicine, Molecular and Cellular Sports Medicine, German Sport University Cologne, 50933 Cologne, Germany

**Keywords:** ultramarathon, sport nutrition, energy metabolism, muscular damage

## Abstract

Background: Ultramarathon running represents an extreme physiological and metabolic challenge. Despite its growing popularity among recreational and competitive runners, evidence-based guidance for nutrition, energy balance, and recovery remains limited. Understanding metabolic response and hormonal regulation during such events is crucial for improving athletes’ health and performance. Methods: This prospective observational study examined participants of the 2024 TorTour de Ruhr^®^ (100 km, 160.9 km, and 230 km). Pre- and post-race assessments included body composition, energy intake and expenditure, metabolic and hormonal biomarkers (leptin, ghrelin, insulin, glucagon, irisin, creatine kinase muscle type (CKM), lactate dehydrogenase (LDH)), and continuous glucose monitoring (CGM). Blood and saliva samples, bioimpedance analysis, and validated symptom questionnaires (General Assessment of Side Effects (GASE)) were used. Results: Of the 43 ultra runners (16 women, 27 men), 39 finished the race: 19 participants of the 100 km group, 8 of the 160.9 km group, and 16 of the 230 km group. Mean energy deficit was 6797 kcal (range: 417–18,364 kcal) with carbohydrate-dominant fueling (79%). Significant reductions in leptin and insulin and increases in ghrelin, glucagon, CKM, and LDH were observed, indicating disrupted energy homeostasis and muscle damage. The 230 km subgroup showed the greatest changes. Gastrointestinal and musculoskeletal symptoms increased post-race, aligning with biomarker patterns. Conclusions: Ultramarathon participation induces profound disturbances in metabolic and structural integrity, regardless of race distance. These findings underline the importance of developing individualized nutritional and recovery strategies and highlight the need for future research to investigate how energy deficit and macronutrient composition interact to influence metabolic strain and post-race recovery.

## 1. Introduction

Ultra-endurance sports, including non-stop ultramarathons, have seen a substantial rise in popularity over the last decade, with thousands of athletes now competing in races that exceed the conventional marathon distance of 42.195 km [1]. These events, often ranging from 50 km to over 230 km and lasting more than 24 h, impose extreme physiological demands, challenging the limits of energy availability, muscle integrity, and metabolic and immune function [2].

Although ultramarathon runners are typically well-trained, numerous studies have shown that these events are associated with significant energy deficits, muscle catabolism, and systemic inflammatory responses, particularly when effective nutritional strategies are lacking [2,3]. During prolonged exertion, energy expenditure far exceeds the amount of energy that can realistically be consumed, resulting in sustained negative energy balance [4]. As such, nutritional planning must go beyond in-race fueling to include pre-race carbohydrate loading, strategic protein timing, and post-race replenishment protocols. Still, evidence-based guidelines for macronutrient composition, optimal intake timing, and in-race tolerability remain lacking for ultra-endurance athletes [5,6]. This leads to inadequate and inconsistent nutrition strategies, resulting in substantial metabolic and inflammatory burden on the body in those athletes [7]. If frequently repeated over time, such large energy deficits may impair endocrine function, compromise bone health, and delay post-race recovery, potentially increasing the risk for long-term performance decline and injury [8].

Despite a growing body of literature, most existing studies focus on short-term outcomes, such as performance metrics or acute changes in body weight. Less attention has been given to the interplay between nutritional behavior and systemic stress, especially in relation to metabolic regulation and muscle damage signaling pathways during actual race conditions. Moreover, ultramarathons differ markedly in structure, duration, and elevation profile—characteristics that are difficult to replicate in laboratory settings. Consequently, findings from controlled laboratory studies often lack ecological validity and may not translate to real-world ultra-endurance performance contexts [9].

A further limitation of current research is the underrepresentation of endocrine markers in the context of ultra-endurance. Hormones such as leptin and ghrelin play a critical role in appetite regulation and energy homeostasis, and their fluctuations during prolonged physical activity may provide insight into central fatigue, fuel prioritization, and post-exercise recovery [10,11]. However, real-world data on these hormones during ultra-endurance races remain scarce. Additionally, the high interindividual variability in physiological and biochemical responses suggests that outcomes are likely influenced by a combination of innate physiological traits (e.g., body composition, hormonal baseline levels) and training-induced reactions (e.g., mitochondrial efficiency, substrate flexibility) [12].

Additionally, recent studies have begun to explore the use of continuous glucose monitoring (CGM) systems to assess real-time glycemic responses during ultra-endurance events [13,14,15]. Although initial data suggest that CGM can capture dynamic fluctuations in interstitial glucose linked to carbohydrate intake and pacing strategy, its application in non-diabetic, ultramarathon settings remains limited and warrants further investigation [16]. Therefore, we monitored interstitial glucose profiles during the race to explore real-time glycemic patterns in relation to distance and nutritional intake.

In our previous study during the TorTour de Ruhr^®^ 2022—one of the longest non-stop ultramarathons in Europe—we demonstrated significant alterations in body composition, inflammatory and metabolic markers, and cardiac stress among athletes who completed the 160.9 km and 230 km distances [17]. However, that study was limited by its focus on the longer distances, the small sample size and a lack of integrated dietary analysis. Building on those findings, the present study aimed to broaden the analytical scope by including all three race distances of the TorTour de Ruhr^®^ 2024 (100 km, 160.9 km, and 230 km) and by incorporating detailed in-race nutritional tracking alongside pre- and post-race biomarker assessments. The goal was to improve how in-race nutritional strategies relate to metabolic and endocrine responses and to help close the existing knowledge gap regarding real-world physiological stress in ultra-endurance competition.

By integrating metabolic, biochemical, and behavioral data under field conditions, this study aims to advance our understanding of how athletes respond to prolonged exertion—and how targeted nutrition strategies may help mitigate its adverse physiological consequences.

The specific aims of this study were to characterize energy expenditure and nutritional intake across different race distances, with a particular focus on macronutrient distribution and overall energy balance. Furthermore, we sought to analyze biochemical markers of muscle damage, including creatine kinase muscle type (CKM) and lactate dehydrogenase (LDH), using pre- and post-race blood and saliva samples. Endocrine regulation of appetite and energy homeostasis was assessed by quantifying circulating leptin and ghrelin concentrations, thereby enabling evaluation of their role in post-race recovery and metabolic response. Finally, we aimed to assess the relationship between nutritional strategies during competition and physiological stress responses, thereby providing a potential basis for developing evidence-based nutritional recommendations for ultra-endurance athletes.

## 2. Methods

### 2.1. Entry Eligibility

TorTour de Ruhr^®^ participation required a medical sports examination that had been conducted less than six months before the race to confirm the physical resilience of the athlete for successful completion of the competition. Additionally, the participants had to provide proof of previous marathon and ultramarathon experience. The registration for the event is by invitation only to ensure entry eligibility.

Inclusion criteria for study participation were as follows: male and female endurance athletes with a minimum age of 18 years, participating in one of the three distances (100 km, 160.9 km or 230 km) of the TorTour de Ruhr^®^ 2024; no previous severe injuries; and the ability to understand the study procedure and to give informed consent.

Exclusion criteria included the following: nicotine consumption; diseases of the intestine; blood clotting disorders or intake of blood-thinning medications; acute or chronic vascular (blood flow) disorders; cardiovascular, metabolic, or autoimmune diseases; and non-consenting subjects.

All subjects received information about the study content and the use of the data and provided written consent. The study was conducted in compliance with the Declaration of Helsinki. The study was approved by the ethics committee of the German Sports University Cologne (protocol code: 012/2024, date of approval: 21 February 2024).

### 2.2. Sample Collection

All measures included pre- and post-assessments. Pre-race measurements were carried out in the evening (230 km) or in the time frame of two hours (100 km and 160.9 km) before the start at the organizer’s race briefing to determine basal resting values, while post-race measurements were performed immediately at the finish line at the finishers’ arrival. The study team was divided into groups responsible for (i) body composition, (ii) laboratory data, and (iii) blood sampling to minimize examination time pre and post. Blood samples were taken pre and post from the *vena mediana cubiti* and anticoagulated using ethylenediaminetetraacetic acid (EDTA) as anticoagulant. Samples were instantly centrifuged at 2000× *g* at 4 °C; the respective supernatant was aliquoted into 2 mL cryotubes and stored at −20 °C until transportation to the analysis facility. All samples were transported in a time frame of a maximum of four hours and either measured immediately in the laboratory or stored at −80 °C until further analysis.

### 2.3. Anthropometry and Body Composition

Anthropometric measurements included height, body mass, and body composition. Height was measured without shoes, in light clothing, with a standardized scale. For measuring body mass and body composition, a bioimpedance scale (InBody 770, InBody Europe B.V., Eschborn, Germany) was used.

### 2.4. Environmental Conditions

Environmental conditions, including pre-testing time points, race starts, and respective weather parameters, are summarized in Table 1.

### 2.5. Energy Intake and Expenditure

Participants received a questionnaire and were asked to note the time of food intake and a detailed description of the respective meal/component. Briefly, the amount of food/snack (in grams/household measurements, i.e., one handful, one bowl, cup, mug, and so on), the fat content for dairy products, the respective brand, all beverages (quantity and brand of beverage, if applicable), and the mixing ratio for homemade drinks should be indicated. Documentation was performed by the mandatory athletes’ team crew or retrospectively by the athlete himself/herself. In addition, the questionnaire included questions regarding gender, age, number of ultramarathons, type/amount/number of supplements, specialized nutritional habits (e.g., vegan/vegetarian), experienced gastrointestinal symptoms related to an ultramarathon, and products that are repeatedly/always used for food during ultramarathons.

For evaluation of the total energy intake and the respective contribution of protein/fat/carbohydrates, the FDDB app (Food Database GmbH, Bremen, Germany) was used. Although FDDB relies on standardized nutrient databases, previous evaluations have shown that such tools provide acceptable accuracy for field-based dietary assessment.

The energy expenditure calculation was based on the heart rate determined by the respective running wearable (Garmin International, Olathe, KS, USA) during the race and the respective energy expenditure data on the Garmin Connect online platform. While wearable-derived estimations of caloric expenditure may show individual variability, heart-rate–based algorithms from Garmin devices have demonstrated reasonable accuracy during prolonged endurance exercise and are widely used in research. Nevertheless, the provided data should be interpreted as approximations rather than precise measurements.

### 2.6. Energy Metabolism and Muscle Damage

Biomarkers for muscle damage—LDH (Merck, Darmstadt, Germany) and CKM (Abcam, Cambridge, UK)—as well as for energy metabolism—leptin, ghrelin (Meso Scale Discovery, Rockville, MD, USA), glucagon-like peptide 1 (GLP-1) (Thermo Fisher Scientific, Waltham, MA, USA), irisin (Phoenix Pharmaceuticals, Burlingame, CA, USA), insulin (Abcam, Cambridge, UK) and glucagon (Thermo Fisher Scientific, Waltham, MA, USA)—were analyzed in the plasma fraction using enzyme-linked immunosorbent assays (ELISA) or multiplex electrochemiluminescence (ECL) immunoassays according to the manufacturers’ instructions.

Tissue glucose was measured with a subcutaneous glucose sensor (FreeStyle Libre 3, analyzed by App-based program LibreView, Abbot Diabetes Care, Alameda, CA, USA). As the number of available devices was limited, sensors were allocated to participants on a voluntary basis and applied successively until the supply was exhausted, resulting in continuous glucose monitoring being conducted in a subgroup of the study cohort, including 10 participants in the 100 km group, 4 in the 160.9 km group, and 3 in the 230 km group.

### 2.7. General Assessment of Side Effects (GASE)

To assess participants’ perceived physical and psychological complaints related to ultra-endurance exertion, we used the General Assessment of Side Effects (GASE) questionnaire [18]. The GASE is a validated self-report instrument originally developed to systematically capture the frequency and intensity of common somatic symptoms and side effects that may occur during medical or physiological stress. Participants were asked to rate the severity of 24 predefined symptoms (e.g., headache, nausea, muscle pain, dizziness, fatigue, gastrointestinal discomfort) on a four-point Likert scale ranging from 0 (not present) to 3 (severe). Additionally, three free-text fields were provided for reporting further complaints not covered by the predefined items. The GASE was administered immediately before (30 to 45 min) and within 15 min after the race to capture acute subjective side effects related to the prolonged physical effort of the race. For analysis, symptom scores were evaluated as total sum scores, with descriptive and comparative statistics stratified by race distance. Furthermore, a single-item descriptive analysis was performed for specific items regarding food intake and processing, as well as items for muscular discomfort and exhaustion.

### 2.8. Statistics

Data analysis was performed using GraphPad Prism (GraphPad Prism 10.5; San Diego, CA, USA). First, for determining the general effect of an ultramarathon on target variables, data from participants of all distances (100 km, 160.9 km and 230 km) were analyzed in one dataset. Secondly, subgroup analysis by race distance was performed for descriptive statistics (anthropometry, energy expenditure/intake, and finish time) and for all other measures. All data were tested on Gaussian distribution using the Kolmogorov–Smirnov normality test. Depending on the target variable and hypothesis, one- or two-tailed t-tests were used comparing pre and post for all normally distributed data. Otherwise, a Wilcoxon matched-pairs signed rank test was used to determine the statistical significance of differences between pre and post. Multiple comparison corrections were consistently applied across all biomarker analyses. Differences between the 100 vs. 160.9 vs. 230 km participants were determined using one-way ANOVA followed by Holm-Šídák’s multiple comparisons test (normally distributed data) or with Kruskal–Wallis test followed by Dunn’s multiple comparisons test (not normally distributed data). If not otherwise stated, all data are presented as mean ± standard deviation. Statistical significance was established at *p* ≤ 0.05.

## 3. Results

### 3.1. Study Group Characteristics

In total, 43 ultramarathon runners (16 f/27 m) were included. Participants had already completed 37 ultramarathons on average. Four participants dropped out during the race. The remaining 39 runners (13 f/26 m) all finished within the defined period (cut-off time: 37 h/230 km, 27 h/160.9 km and 17 h/100 km). Data of all finishers were used for analysis. Detailed pre-race anthropometric characteristics of the runners from the different distances are presented in Table 2. Additionally, post-race body mass was recorded; however, reliable bioimpedance body composition assessment could not be performed for all participants due to environmental conditions and the exhausted physical state of the runners at the finish line (for details, see Supplementary Table S1).

### 3.2. Energy Intake and Expenditure

Mean total energy intake increased with race distance; however, it remained consistently below the estimated energy expenditure across all groups. Consequently, participants did not meet the caloric demands of the race, with those competing in 230 km exhibiting proportionally greater energy deficits compared to 160.9 km and 100 km athletes. Detailed data from the energy intake and expenditure, as well as fluid intake of the runners from the different distances, are presented in Table 3.

### 3.3. Macronutrient Distribution

Macronutrient analysis demonstrated a clear predominance of carbohydrate-based fueling strategies across all race distances (78.64 ± 0.17% of total energy intake) (see Table 4). On average, carbohydrates contributed the largest proportion to total energy intake, primarily in the form of rapidly absorbable sources such as gels, bars, and isotonic beverages. Protein intake remained relatively low consistently, accounting for less than 10% of total caloric intake in all groups (8.9 ± 0.05% of total energy intake). Fat contributed 12.46 ± 0.12% of total energy intake. It should be emphasized that the relative distribution of macronutrients did not differ substantially between the three distances. However, considerable interindividual variability was observed in both nutrient timing and food selection, particularly among participants in the longer-distance categories.

### 3.4. Continuous Glucose Monitors

Interstitial glucose levels during the race were continuously monitored in a subgroup of 17 participants using a CGM system (FreeStyle Libre 3, Abbot Diabetes Care, Alameda, CA, USA). Mean glucose concentrations varied slightly between distance groups but remained within the normoglycemic range across all athletes (see Table 5). Participants in the 100 km group displayed the highest average glucose levels (120.4 ± 15.2 mg/dL), followed by those in the 230 km (113.4 ± 5.7 mg/dL) and 160.9 km groups (108.3 ± 10.9 mg/dL).

Notably, no cases of clinically relevant hypoglycemia (<70 mg/dL) were observed during the monitored race periods.

### 3.5. Energy Metabolism

**Figure 1:**
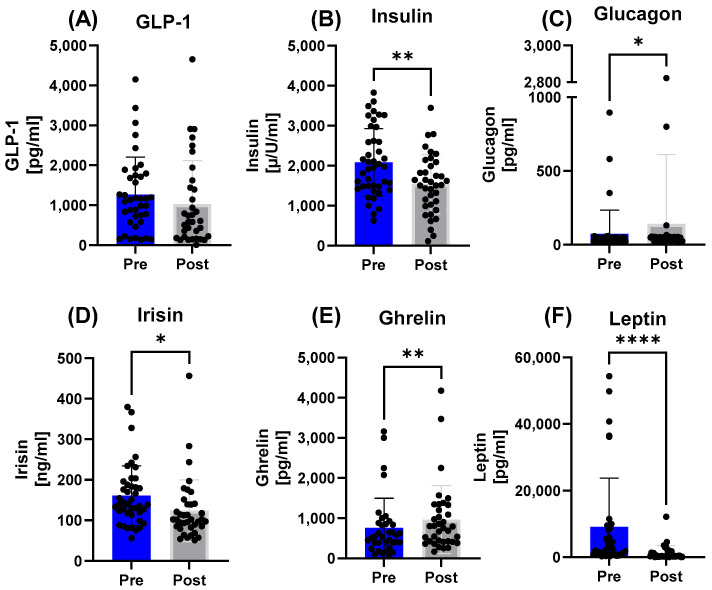
Endocrine markers of energy metabolism in the total study population before and after the race. Changes in (**A**) GLP-1, (**B**) insulin, (**C**) glucagon, (**D**) irisin, (**E**) ghrelin, and (**F**) leptin are shown. Significant alterations were observed for leptin, ghrelin, irisin, insulin, and glucagon. Note: * = *p* ≤ 0.05, ** = *p* ≤ 0.01, **** = *p* ≤ 0.0001.

Analysis of endocrine markers related to energy metabolism in the overall study population showed significant changes from pre- to post-race (Figure 1). Circulating leptin levels decreased (*p* < 0.0001), while ghrelin levels increased (*p* = 0.0083). Irisin also showed a rise post-race (*p* = 0.0160). In contrast, changes in GLP-1 did not reach statistical significance (*p* = 0.1396). Regarding hormones of glucose metabolism, insulin decreased (*p* = 0.0033), whereas glucagon increased (*p* = 0.0139).

**Figure 2:**
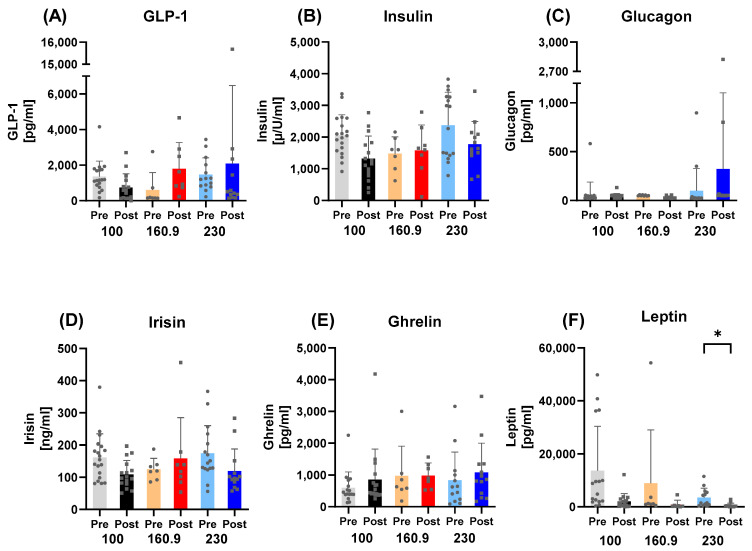
Endocrine markers of energy metabolism by race distance (100 km, 160.9 km, 230 km). Changes in (**A**) GLP-1, (**B**) insulin, (**C**) glucagon, (**D**) irisin, (**E**) ghrelin, and (**F**) leptin are shown. GLP-1, insulin, glucagon, irisin, and ghrelin showed no significant subgroup differences, whereas leptin decreased significantly in the 230 km group but not in the 160.9 km or 100 km groups. Note: * = *p* ≤ 0.05.

Subgroup analyses by race distance revealed that leptin was significantly reduced in the 230 km group (*p* = 0.0289), but no significant differences were detected in the 160.9 km (*p* = 0.7608) or 100 km (*p* = 0.0681) subgroups. Ghrelin did not change significantly in the 230 km (*p* > 0.9999), 160.9 km (*p* > 0.9999), or 100 km (*p* > 0.9999) groups. Similarly, irisin remained unchanged in the 230 km (*p* = 0.3525), 160.9 km (*p* = 0.9496), and 100 km (*p* = 0.3162) subgroups. For GLP-1, no significant differences were observed in the 230 km (*p* = 0.9655), 160.9 km (*p* = 0.8498), or 100 km (*p* = 0.9461) groups. Insulin did not change significantly in the 230 km (*p* = 0.3069) or 160.9 km (*p* = 0.9998) groups, while a trend toward reduction was observed in the 100 km subgroup (*p* = 0.0683). Glucagon did not differ significantly in the 230 km (*p* = 0.4839), 160.9 km (*p* > 0.9999), or 100 km (*p* > 0.9999) subgroups (Figure 2).

### 3.6. Muscle Damage

**Figure 3:**
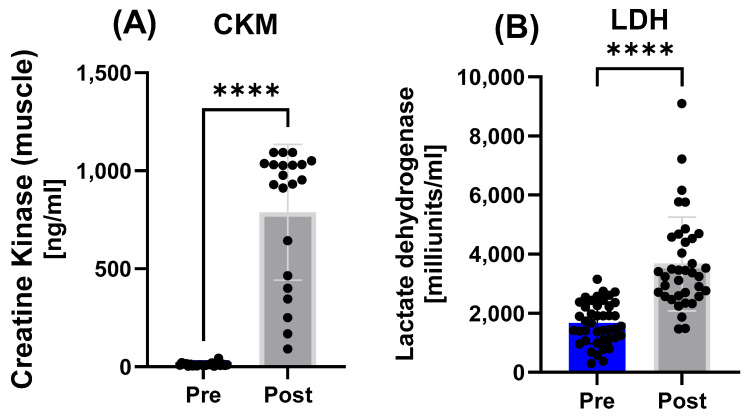
Markers of muscle damage in the total study population before and after the race. Significant increases were observed for (**A**) CKM and (**B**) LDH. Note: **** = *p* ≤ 0.0001.

Biomarkers of muscle damage showed elevations in the overall cohort (Figure 3). CKM increased significantly (*p* < 0.0001), as well as LDH (*p* < 0.0001).

**Figure 4:**
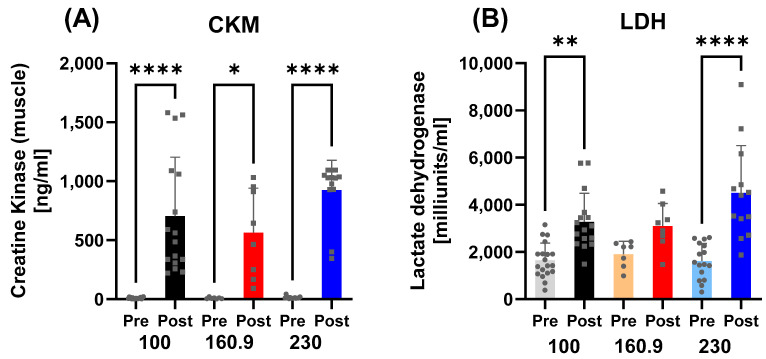
Markers of muscle damage by race distance (100 km, 160.9 km, 230 km). (**A**) CKM increased significantly in all distance groups. (**B**) LDH increased in the 230 km and 100 km groups, but not in the 160.9 km group. Note: * = *p* ≤ 0.05, ** = *p* ≤ 0.01, **** = *p* ≤ 0.0001.

Subgroup analyses confirmed an increase in CKM across all distances (230 km: *p* < 0.0001; 160.9 km: *p* = 0.0278; 100 km: *p* < 0.0001). For LDH, significant increases were observed in the 230 km (*p* < 0.0001) and 100 km (*p* = 0.0011) subgroups, whereas no significant change was found in the 160.9 km group (*p* = 0.3546) (Figure 4).

### 3.7. GASE Score

**Figure 5:**
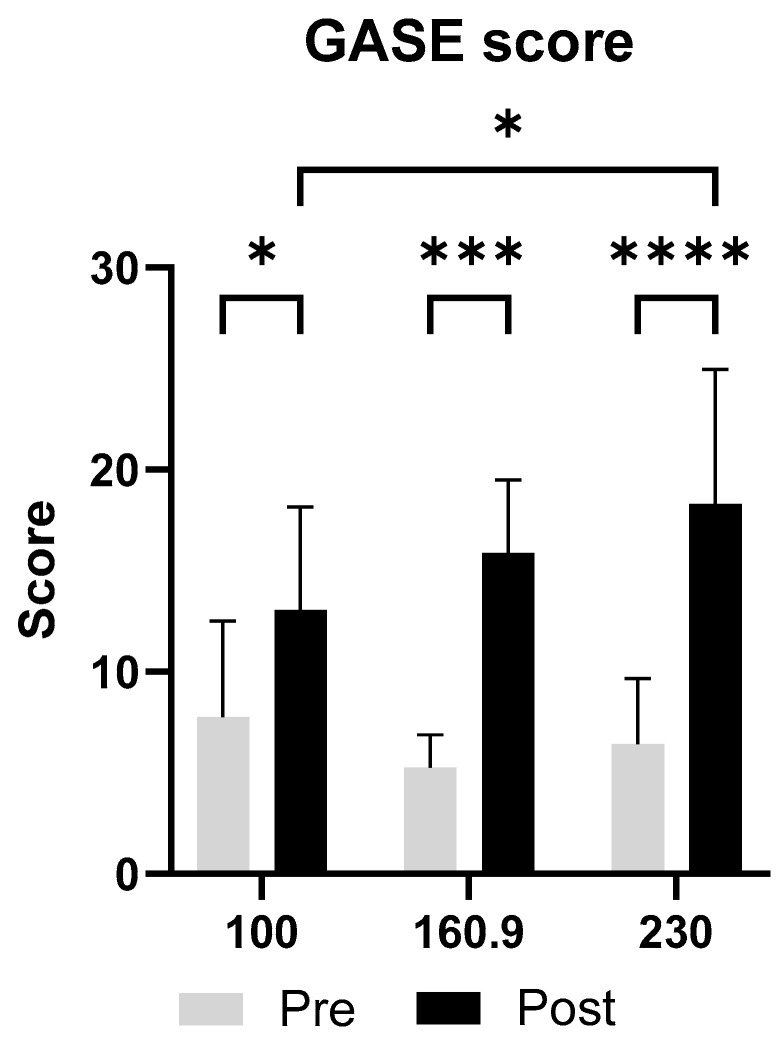
Comparison of sum scores of the GASE questionnaire before and after the race across three race distances (100 km, 160.9 km, 230 km). Mean ± SD sum scores derived from the GASE questionnaire are shown for each race distance pre- and post-race. Across all distances, total symptom burden increased after race completion. The largest post-race values were observed in the 230 km group. Additionally, the post-race sum score of the longest running distance (230 km) was significantly elevated compared to the post-race score of the shortest distance (100 km). Note: * = *p* ≤ 0.05, *** = *p* ≤ 0.001, **** = *p* ≤ 0.0001.

Analysis of the GASE questionnaire exposed that total symptom burden, expressed as the sum score, increased from pre- to post-race across all distances (Figure 5). The 230 km group showed the largest increase in the mean sum score from 6.4 ± 3.3 before the race to 18.3 ± 6.6 after the race (*p* < 0.001). In the 160.9 km group, values increased from 5.3 ± 1.6 to 15.9 ± 3.6 (*p* = 0.003), while the 100 km group showed an increase from 7.8 ± 4.8 to 13.1 ± 5.1 (*p* = 0.0202). When comparing the post-race scores of the different distances, a significant increase from the 100 km group to the 230 km group was detected (*p* = 0.0400).

**Figure 6:**
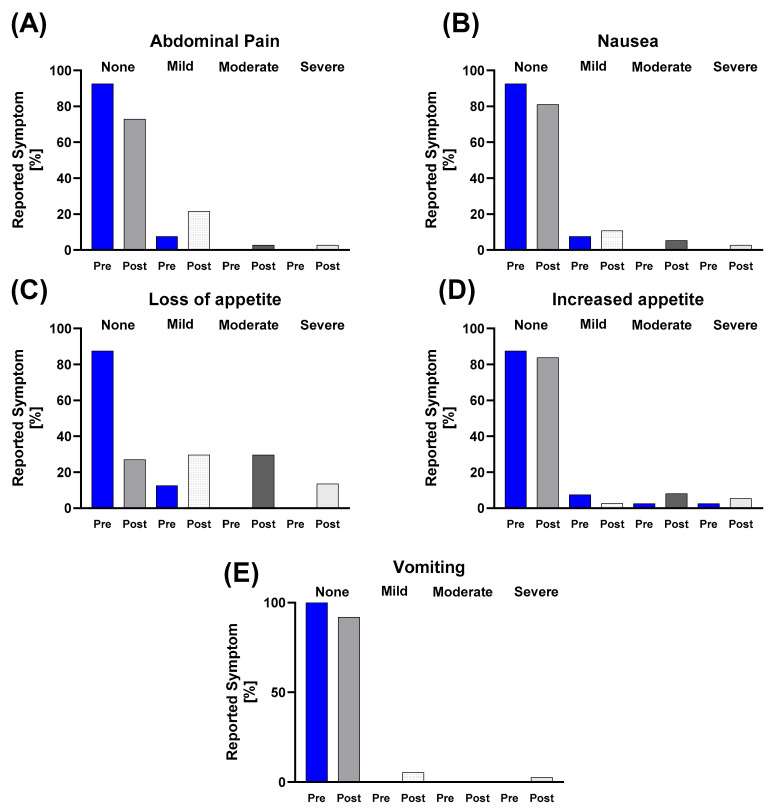
Descriptive analysis of prevalence and severity of nutrition-related symptoms from the GASE questionnaire before and after the race in the total study population. Charts depict the relative frequency (%) of (**A**) abdominal pain, (**B**) nausea, (**C**) loss of appetite, (**D**) increased appetite and (**E**) vomiting, categorized by symptom severity (none, mild, moderate, severe). The post-race period was characterized by an increase in prevalence and severity of all gastrointestinal complaints, particularly loss of appetite.

When examining nutrition-related symptoms across the total study population, abdominal pain was absent in 92.5% of participants pre-race, with only 7.5% reporting mild symptoms; post-race, the percentage without symptoms dropped to 73.0%, while 21.6% reported mild, 2.7% moderate, and 2.7% severe symptoms. Nausea showed a similar pattern, increasing from 7.5% mild pre-race to 10.8% mild, 5.4% moderate, and 2.7% severe post-race. Vomiting, which was absent pre-race, was reported post-race by 5.4% with mild and 2.7% severe symptoms. Loss of appetite increased from 12.5% mild pre-race to 29.7% mild, 29.7% moderate, and 13.5% severe post-race. Increased appetite pre-race was reported as 7.5% mild, 2.5% moderate and 2.5% severe, changing to 2.7% mild, 8.1% moderate, and 5.4% severe post-race (Figure 6).

**Figure 7:**
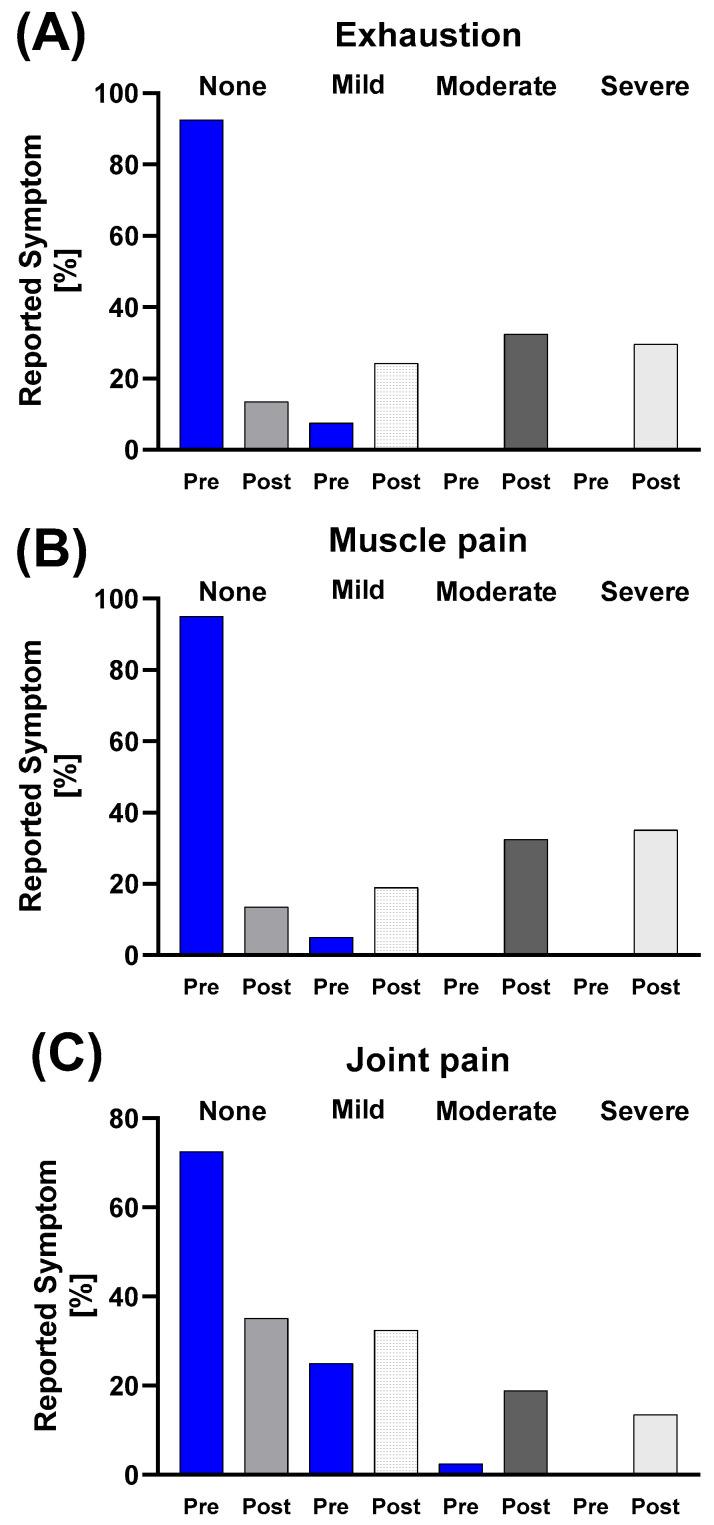
Descriptive analysis of prevalence and severity of muscle-related symptoms from the GASE questionnaire before and after the race in the total study population. Charts depict the relative frequency (%) of (**A**) exhaustion, (**B**) muscle pain, and (**C**) joint pain, categorized by symptom severity (none, mild, moderate, severe). Post-race data showed a shift toward higher severity categories, with exhaustion and muscle pain affecting almost all participants.

Muscle-related symptoms showed more pronounced increases. Exhaustion was absent in 92.5% pre-race, with 7.5% reporting mild symptoms; post-race, only 13.5% reported no symptoms, while 24.3% reported mild, 32.4% moderate, and 29.7% severe symptoms. Muscle pain increased from 5% mild pre-race to 18.9% mild, 32.4% moderate, and 35.1% severe post-race. Joint pain increased from 25% mild and 2.5% moderate pre-race to 32.4% mild, 18.9% moderate, and 13.5% severe post-race (Figure 7).

## 4. Discussion

Ultramarathon running imposes an exceptional metabolic burden that exceeds conventional endurance events [19], requiring coordinated reactions in energy balance, endocrine regulation, and musculoskeletal integrity. In the present study, we demonstrated that severe energy deficits were accompanied by marked endocrine responses, evidence of muscle damage, and increased gastrointestinal and musculoskeletal complaints, with the strongest responses observed in the longest race distance. These findings highlight the complex physiological challenges of ultra-endurance exercise and underscore the need for tailored nutritional and recovery strategies.

### 4.1. Energy Homeostasis: Energy Deficit, Hormonal Responses, and Glucose Dynamics

A central finding of this study was the substantial caloric deficit observed across all distances, which progressively increased with race distance. Despite carbohydrate-dominant fueling strategies (~79% of intake), energy intake remained insufficient to meet the exceptionally high energy requirements. This mismatch between energy consumption and demand is consistent with previous ultramarathon research [2,4], where the physical and logistical constraints of ingesting and absorbing sufficient calories under prolonged exertion result in persistent negative energy balance.

The examined endocrine markers also reflected these challenges of energy metabolism. Leptin, secreted by adipocytes as well as by the stomach in response to food intake, acts as a signal of energy sufficiency and regulates appetite and energy expenditure [20]. Its pronounced decrease in our cohort, particularly in the 230 km group, indicates acute depletion of energy reserves and may predispose athletes to post-race hyperphagia and alterations in recovery processes [21]. In line with our findings, previous work has shown that severe energy deficits during strenuous physical activity are accompanied by marked reductions in circulating leptin concentrations, reflecting the tight connection between energy availability and appetite regulation [21]. Similarly, field observations in ultramarathon running consistently report pronounced decreases in leptin across prolonged events, highlighting its role as a sensitive marker of acute energy imbalance in real-world ultra-endurance settings [2].

Conversely, ghrelin, an orexigenic hormone produced in the stomach, increased significantly in the overall population, reflecting compensatory hunger signaling in response to caloric deficit [22]. In contrast to our results, several previous studies have reported an acute suppression of ghrelin following endurance exercise, a phenomenon commonly referred to as exercise-induced anorexia [23,24]. The apparent discrepancy may be explained by differences in study design, exercise duration, or methodological aspects of ghrelin assessment, as only total ghrelin was measured in this study. Since acylated ghrelin, rather than total ghrelin, exerts the primary orexigenic effect, its specific assessment may be required to fully capture exercise-induced changes in appetite regulation [24]. This complexity, together with the relatively small sample size in our subgroup analyses, may explain why no differences across distances were detected in the present study.

Insulin decreased significantly across the total cohort, while glucagon levels increased. This reciprocal hormonal shift is well in line with established models of prolonged exercise [25]. Suppression of insulin together with elevations in glucagon promotes hepatic glucose production and mobilization of alternative substrates such as free fatty acids, consequently ensuring the maintenance of euglycemia despite limited exogenous supply [25]. These endocrine reactions reflect a coordinated strategy to preserve glucose availability for the central nervous system and working muscles, while simultaneously increasing reliance on lipid oxidation to sustain energy demands over many hours.

In line with this, CGM data demonstrated that interstitial glucose concentrations remained within the normoglycemic range across all race distances, with no episodes of clinically relevant hypoglycemia. This stability, despite severe energy deficits, underscores the robustness of glucose regulatory mechanisms under extreme stress [26]. Previous work using CGM in athletes similarly reported that glucose homeostasis can be maintained even in prolonged endurance exercise [13,14,15], though it should be emphasized that stable glucose does not equate to adequate energy availability, as reflected by the endocrine disruptions observed in this study.

Irisin, a myokine secreted in response to muscular contraction, also increased post-race. This observation is noteworthy, as irisin has been linked to the browning of white adipose tissue and enhanced energy expenditure, providing a potential mechanism for adaptive remodeling of energy metabolism in response to repeated endurance stress [27]. While acute increases in irisin have been reported following both resistance and endurance exercise, the magnitude and duration of the response during ultra-endurance competition remain poorly understood [28]. Our findings could suggest that extreme muscular work, such as in ultramarathon running, may serve as a potent stimulus for irisin release, potentially contributing to longer-term metabolic reactions in highly trained athletes.

In contrast, GLP-1, an incretin hormone that enhances insulin secretion and regulates appetite, did not show significant changes across the study population. This is in line with other observations reporting that incretin responses during ultra-endurance exercise are less pronounced [29]. Since GLP-1 also plays a role in satiety regulation, its lack of substantial alteration suggests that while it may contribute to appetite disturbances, the pronounced gastrointestinal complaints observed in our cohort are likely driven by additional factors, like peripheral and mechanical stressors, beyond incretin signaling.

The stability of interstitial glucose concentrations observed via CGM highlights the robustness of glucose regulation despite severe energy deficits, while the absence of significant changes in GLP-1 underlines that incretin pathways may play only a minor role in acute ultra-endurance exertion. The lack of clear differences for some markers, such as ghrelin or GLP-1, points to substantial interindividual variability that may override distance effects. This integrated pattern of classical and emerging biomarkers underscores the multifaceted endocrine adjustments required to sustain performance under extreme metabolic stress and provides a framework for better understanding nutrition and recovery strategies in ultra-endurance athletes.

Taken together, our findings demonstrate that ultramarathon running imposes a profound disturbance of energy homeostasis, reflected not only by the marked caloric deficits and reductions in leptin but also by compensatory increases in ghrelin, the reciprocal insulin–glucagon shift maintaining glucose availability, and the rise in irisin.

### 4.2. Muscle Damage and Structural Stress

Biomarkers of muscle damage revealed pronounced increases in CKM and LDH, consistent with exercise-induced muscle fiber disruption and leakage of intracellular enzymes into circulation [30,31,32,33]. These elevations were particularly marked in the 230 km subgroup but were also significant in 100 km and 160.9 km runners, indicating that even “shorter” ultramarathons impose considerable muscle stress. CKM, a sensitive and well-established marker of skeletal muscle injury [2], showed the strongest response in our cohort, with marked elevations in all subgroups. Such increases reflect structural stress on muscle fibers caused by repetitive eccentric loading, prolonged contractile activity, and mechanical strain during running. While LDH showed no significant increase in the 160.9 km group, its elevation in the 230 km and 100 km groups highlights the heterogeneity of muscle damage responses, potentially linked to differences in pacing, running economy, or terrain exposure.

These findings verify earlier reports showing that prolonged ultramarathon participation leads to substantial muscle damage, which may impair recovery and contribute to long-term musculoskeletal strain if repeated frequently [34,35]. The marked CKM and LDH elevations emphasize the need for adequate recovery periods and monitoring strategies to reduce the risk of overuse injuries. In this context, integrating biochemical monitoring into athlete support may help to individualize recovery and nutritional interventions, thereby improving performance sustainability and reducing injury risk long-term.

### 4.3. Symptom Burden and Its Integration with Biological Stress Markers

The GASE questionnaire provided valuable insights into subjective health burden and allowed integration with biomarker data. Gastrointestinal complaints were frequent, with nausea, abdominal pain, and appetite loss markedly increasing post-race. These findings are consistent with the high prevalence of gastrointestinal distress in ultramarathons [36], which is often linked to reduced splanchnic blood flow, altered gut permeability, and high carbohydrate intake under stress [37]. The strong increase in loss of appetite post-race is of particular concern, as it may further hinder post-exercise recovery by delaying replenishment of glycogen and protein stores. This is further supported by our biomarker analysis, which showed significant reductions in leptin and elevations in ghrelin across the total cohort, indicating a mismatch between energy availability and hunger signaling after the race.

Musculoskeletal complaints, including exhaustion, muscle pain, and joint pain, also increased sharply after the race, affecting nearly all athletes. These subjective reports correspond well with the biochemical evidence of muscle damage, as reflected in significant increases in CKM and LDH across the overall cohort. This alignment underscores that structural stress during ultra-endurance exercise is both objectively measurable and subjectively perceived by the athletes.

Importantly, the integration of subjective (GASE) and objective (biomarkers) data highlights that ultramarathon participation imposes a multidimensional strain on the body, with gastrointestinal and musculoskeletal systems most affected.

### 4.4. Practical Implications

From an applied perspective, our findings highlight the urgent need for nutritional strategies that extend beyond the traditional focus on in-race carbohydrate provision. While carbohydrates remain essential for sustaining high-intensity efforts, relying almost exclusively on rapidly available sources can leave athletes vulnerable to effects of enormous energy deficits, gastrointestinal distress, and impaired recovery [38]. Pre-race, structured carbohydrate loading remains crucial, but sufficient baseline protein intake may also support muscle resilience and repair [4]. In-race, modest protein ingestion (e.g., amino acid–enriched beverages or protein-containing sports foods) has been suggested to attenuate muscle breakdown in prolonged endurance events [39,40]. Post-race, protein becomes even more critical, with recommendations of ≥1.6–2.0 g/kg/day to optimize glycogen resynthesis and muscle recovery [7,38]. Combining carbohydrates with protein appears particularly beneficial in this regard [41].

Regarding CGM, while our data confirms feasibility, its added value for guiding non-diabetic ultrarunners seems limited. Rather than enabling real-time adjustments, CGM may currently serve better as a research tool to characterize feeding patterns and glucose dynamics, but its application requires further validation.

Nevertheless, the intake of fat needs to be considered, as previous research was able to demonstrate that with increasing race distances, higher intake of fat may be critical for success [42]. The prolonged durations and relatively slower running speeds that characterize ultra-endurance events enable increased rates of fat oxidation for long-term energy production [4]. To summarize, a greater tolerance for both carbohydrates and fats, along with sufficient protein intake, is likely necessary for the successful completion of an ultramarathon. This process should be routinely integrated into daily training sessions to promote familiarity and efficacy. This, in turn, will facilitate the gastrointestinal system’s reaction to the strain of larger intake and minimize gastrointestinal distress.

### 4.5. Strengths and Limitations

The primary strength of this study is its comprehensive approach: combining biochemical markers of energy metabolism and muscle damage with real-time glucose monitoring and subjective assessments of gastrointestinal and musculoskeletal complaints. This versatile design, together with subgroup analyses across three distances of the same event, allowed for a unique comparison between different distances under identical environmental conditions. This approach enhances the meaningfulness of our study and enables sophisticated conclusions regarding distance-dependent physiological responses.

Nevertheless, several limitations must be considered. First, although biomarker dynamics were assessed pre- and post-race, it was not possible to conduct follow-up measurements in the hours to days following completion. Such data would be highly informative for understanding the recovery kinetics of endocrine responses. Second, while food intake was carefully documented, reliance on self-reports or reports by the mandatory athletes’ crew may have underestimated or misclassified actual consumption, particularly during the night or in the later stages of the race. Third, reliable post-race body composition assessment via bioimpedance was not feasible due to adverse weather, logistical constraints, and the exhausted state of the athletes, and because acute fluid shifts and gastric filling led to physiologically implausible values that precluded meaningful interpretation. Consequently, hydration status and body mass loss—which may affect heart-rate-based EE estimation—could not be systematically quantified. This represents a limitation, as HR-derived EE may be influenced by hydration-related changes. Nevertheless, such data would have been valuable to more precisely assess the acute impact of the caloric deficits on muscle and fat mass. Fourth, as the race route was characterized by a relatively flat elevation profile, our findings—particularly regarding muscle damage—cannot be directly extrapolated to ultramarathons with substantial elevation gain, where eccentric loading might play an even greater role. Fifth, while we used heart-rate–based EE estimates derived from Garmin running watches, recent studies of Garmin devices indicate considerable interindividual differences in EE estimation [43]. Thus, our EE data should be regarded as approximations and are most reliable for between-group comparisons rather than exact quantification. Finally, no a priori sample size calculation was performed due to the event-based nature of the study, and the resulting limited subgroup sample sizes—particularly in the 160.9 km group—likely reduced statistical power for detecting subtle effects and limited the generalizability of the findings.

## 5. Conclusions

This study demonstrates that ultramarathon running induces severe energy deficits, endocrine disruptions, and muscle damage, accompanied by a substantial burden of gastrointestinal and musculoskeletal symptoms. These effects increase with distance, with the 230 km subgroup exhibiting the most pronounced alterations. In contrast to standardized endurance events such as marathons, ultramarathons include highly variable formats in distance, terrain, and environmental demands, underscoring that uniform nutritional or recovery recommendations are unlikely to be sufficient. Instead, individualized and professionalized approaches are required, particularly as participation increasingly includes recreational athletes who may be more vulnerable to undesirable responses and long-term health risks. While our findings confirm stable glucose homeostasis despite severe deficits, the observed endocrine and muscular changes highlight that profound metabolic and structural stress persists. Importantly, these results can inform athletes, coaches, and race organizations in developing evidence-based fueling and recovery strategies tailored to race distance and individual metabolic responses. By recognizing the considerable physiological burden associated with extreme energy deficits, athletes may be better able to prevent maladaptive responses, improve post-race regeneration, and reduce the risk of long-term health consequences. Future studies should therefore not only refine nutritional and recovery strategies but also include repeated intra-race biomarker assessments to capture the kinetics of these responses. Determining potential physiological thresholds across different ultramarathon distances—at which metabolic and structural strain increases disproportionately—may be critical for developing targeted interventions to optimize performance and safeguard long-term athlete health.

## Figures and Tables

**Table 1:** Pre-testing schedule, race start, and environmental conditions across the three distances of the TorTour de Ruhr 2024.

Variables	100 km	160.9 km	230 km
Pre-testing	19 May 2024 2–3 a.m.	18 May 2024 4–5:30 p.m.	17 May 2024 6–8 p.m.
Start of race	19 May 2024 4 a.m.	18 May 2024 6 p.m.	18 May 2024 8 a.m.
Start conditions	7.4 °C 80% humidity 14.7 km/h south wind	21.5 °C 66% humidity 7.8 km/h west wind	9.2 °C 85% humidity 10.5 km/h west wind
End of race	19 May 2024 10:30 a.m.–9:00 p.m.;		
Finish conditions	Mean 14 °C, 83% humidity, heavy rain at times, 14 km/h west wind		

Notes: Values indicate the timing of pre-race assessments, race start, and environmental conditions for each race distance. Pre-testing time points refer to baseline measurements conducted prior to race initiation. Start and finish conditions include ambient temperature (degrees Celsius, °C), relative humidity (percentage, %), and wind speed/direction (kilometers per hour, km/h).

**Table 2:** Anthropometric data and finish time of the study participants subdivided into each race distance. The anthropometric data presented was collected pre-race. All data are presented as mean ± standard deviation (SD).

Variables	100 km	160.9 km	230 km
Finisher (Drop-outs)	n = 18 (n = 1)	n = 7 (n = 1)	n = 14 (n = 2)
Sex	Female: n = 10 Male: n = 9	Female: n = 1 Male: n = 7	Female: n = 5 Male: n = 11
Age [years]	51.6 ± 9.1	48.0 ± 7.7	47.2 ± 7.7
Body mass [kg]	70.6 ± 8.5	74.8 ± 7.7	71.2 ± 12.7
Body fat mass [kg]	15.3 ± 4.2	13.6 ± 4.6	10.8 ± 4.5
Whole Body Phase Angle 50 kHz [°]	5.4 ± 0.6	5.9 ± 0.7	5.7 ± 0.6
Finish time [h]	14.3 ± 1.8	22.5 ± 2.1	32.5 ± 2.9
Running distance per week [km]	59.3 ± 22.6	72.5 ± 36.1	85.3 ± 14.4
Mean finished marathons	49.3 ± 52.7	46.6 ± 36.7	54.7 ± 55.1
Mean finished ultramarathons	27.7 ± 37.4	32.8 ± 28.2	49.4 ± 48.6

Notes: The anthropometric data presented was collected pre-race. All data are presented as mean ± standard deviation (SD). Finishers (drop-outs) are reported as absolute numbers. Age (years); body mass (kilograms, kg); body fat mass (kilograms, kg); whole body phase angle at 50 kHz (degrees, °); finish time (hours, h); running distance per week (kilometers, km). Numbers of finished marathons and ultramarathons reflect lifetime participation.

**Table 3:** Energy balance and fluid intake data of study participants across the three race distances. Energy intake, expenditure and fluid intake are presented as mean ± SD. Energy deficit is reported as mean ± SD, along with the minimum and maximum observed values (range).

Variables	100 km	160.9 km	230 km
Energy Expenditure [kcal]	7401.7 ± 1730.2	11,420.3 ± 535.6	14,634.6 ± 4784.4
Energy Intake [kcal]	2453.6 ± 1169.1	4234.9 ± 2486.5	6042.3 ± 2632.9
Energy Deficit [kcal]	5220.7 ± 2600.9 (range: 1003–10258)	7066.4 ± 2577.9 (range: 2259–9555)	8562.3 ± 4688.1 (range: 417–18,364)
Fluid Intake [mL]	4149.0 ± 1844.2	5955.6 ± 5956	11,936.1 ± 8117.7

Notes: Energy intake, expenditure, and fluid intake are presented as mean ± standard deviation (SD). Energy deficit is reported as mean ± SD and includes the observed minimum and maximum values (range). Energy expenditure (kilocalories, kcal); energy intake (kilocalories, kcal); energy deficit (kilocalories, kcal); fluid intake (milliliters, mL).

**Table 4:** Macronutrient distribution data of all study participants. Respective macronutrients are presented as mean percentage ± SD of the total energy intake.

Macronutrient	Mean% of Total Energy Intake (All Athletes)
Fats [%]	12.46 ± 0.1
Carbohydrates [%]	78.64 ± 0.2
Proteins [%]	8.90 ± 0.1

Notes: Macronutrient data are presented as mean percentage ± standard deviation (SD) of total energy intake. Fats (percent of total energy intake, %); carbohydrates (percent of total energy intake, %); proteins (percent of total energy intake, %).

**Table 5:** Continuous glucose monitor values of study participants across three distances. Respective interstitial glucose values are presented as mean ± SD, and relative glucose variability as percentage, along with the minimum and maximum observed values (range).

Variables	100 km	160.9 km	230 km
Runners	n = 10	n = 4	n = 3
Interstitial Glucose [mg/dl]	120.4 ± 15.2	108.3 ± 10.9	113.4 ± 5.7
Relative Glucose Variability [%]	12.6	10.0	5.0
Range [mg/dl]	77–218	70–175	82–153

Notes: Interstitial glucose values are presented as mean ± standard deviation (SD). Relative glucose variability is expressed as a percentage (%). The glucose range is reported as minimum to maximum observed values (milligrams per deciliter, mg/dL). Interstitial glucose (milligrams per deciliter, mg/dL); relative glucose variability (percentage, %); glucose range (milligrams per deciliter, mg/dL).

## Data Availability

All data that are not provided in the main manuscript can be provided by the corresponding author upon reasonable request. Not all data are publicly available owing to privacy restrictions.

## Supplementary Materials

The following supporting information can be downloaded at: https://www.mdpi.com/article/10.3390/nu17233801/s1, Table S1: Anthropometric data before (Pre) and after (Post) the race of the study participants subdivided into each race distance. All data are presented as mean ± standard deviation (SD).
