# Supplementary material for: Does Distance Matter? Metabolic and Muscular Challenges of a Non-Stop Ultramarathon with Sub-Analysis Depending on Running Distance
Source: Nutrients. 2025 Dec 4;17(23):3801. doi: 10.3390/nu17233801 (PMC12693733; doi:10.3390/nu17233801)
Supplement: Supplementary file 1 [file nutrients-17-03801-s001.zip › nutrients-3985384-supplementary.pdf]

Supplementary Table S1: Anthropometric data before (Pre) and after (Post) the race of the study participants subdivided into each race distance. All data are presented as mean ± standard deviation (SD).

Variables	100 km		160.9 km		230 km	
**Time of measurement**	Pre<br>n = 14	Pos<br>n = 12t	Pre<br>n = 7	Post<br>n = 7	Pre<br>n = 15t	Post<br>n = 11t
**Body mass [kg]**	70.6 ± 8.5	71.4 ± 8.2	74.8 ± 7.7	73.4 ± 7.6	71.2 ± 12.7	73.1 ± 14
**Body fat mass [kg]**	15.3 ± 4.2	14.8 ± 5	13.6 ± 4.6	13.1 ± 4.5	10.8 ± 4.5	10.4 ± 4.5
**Skeletal muscle mass [kg]**	30.7 ± 4.2	31.7 ± 4.7	34.5 ± 5.5	34 ± 5.3	33.9 ± 5.8	32.2 ± 11.7
**Total body water [L]**	40.6 ± 5.4	41.5 ± 5.9	44.9 ± 6.8	44.2 ± 6.4	44.4 ± 7.2	46 ± 8.7
